# Correction: Relationship between the Uncompensated Price Elasticity and the Income Elasticity of Demand under Conditions of Additive Preferences

**DOI:** 10.1371/journal.pone.0154487

**Published:** 2016-04-21

**Authors:** Lorenzo Sabatelli

The following information is missing from the Funding section: The author also received partial financial support from the Bill and Melinda Gates Foundation through the Disease Control Priorities Project (Department of Global Health of the University of Washington, Seattle).

In the Methods section, there are errors in the first and fifteenth equations. In the subsection “Relationship between Income Elasticity and Price Elasticity of Demand,” the variable ϖ in Eq 1 should read ω. Please view the complete, correct equation here:

$$\eta_{i}=-\frac{1}{\rho }\omega_{i}\varepsilon_{i}{}^{2}+\left(\frac{1}{\rho }-\omega_{i}\right)\varepsilon_{i}$$ (1)

In the subsection “Step 2,” the variable ϖ in Eq 15 should read ω. Please view the complete, correct equation here:

$$\varepsilon_{i}=\frac{d\left(\text{log}\;q_{i}\right)}{d\left(\text{log}\;E\right)}=\frac{\theta_{i}}{\omega_{i}}$$ (15)
