# Supplementary material for: Relationship between the Uncompensated Price Elasticity and the Income Elasticity of Demand under Conditions of Additive Preferences
Source: PLoS One. 2016 Mar 21;11(3):e0151390. doi: 10.1371/journal.pone.0151390 (PMC4801373; doi:10.1371/journal.pone.0151390)
Supplement: S1 Fig — Sources: Santerre & Vernon 2006, Seale et al. 2003, Ringel et al. 2002, & Manning et al. 1987. The green area highlights the 95% credible interval (CrI) of the simulated values of the price elasticity of demand. The red rectangles refer to specific empirical studies conducted in the US. The white rectangles refer to global estimates, based on survey data, produced by the US Department of Agriculture (Seale et al., 2003). The data lying within the 95% CrI is considered consistent with model predictions.The average budget share was drawn from a uniform distribution ranging from 0.0001 to 0.1, and the elasticity of the marginal utility of income was drawn from a normal distribution with mean equal to -1.26 and standard deviation equal to 0.1. (DOCX) [file pone.0151390.s001.docx]

**Relationship Between the Uncompensated Price Elasticity and the Income Elasticity of Demand Under Conditions of Additive Preferences**

Author: Lorenzo Sabatelli, Ph.D

Author affiliation: GLOBMOD Health, Market Analysis Unit, Barcelona, Spain

Correspondence: Lorenzo.Sabatelli@globmod.com

**Supporting figure**


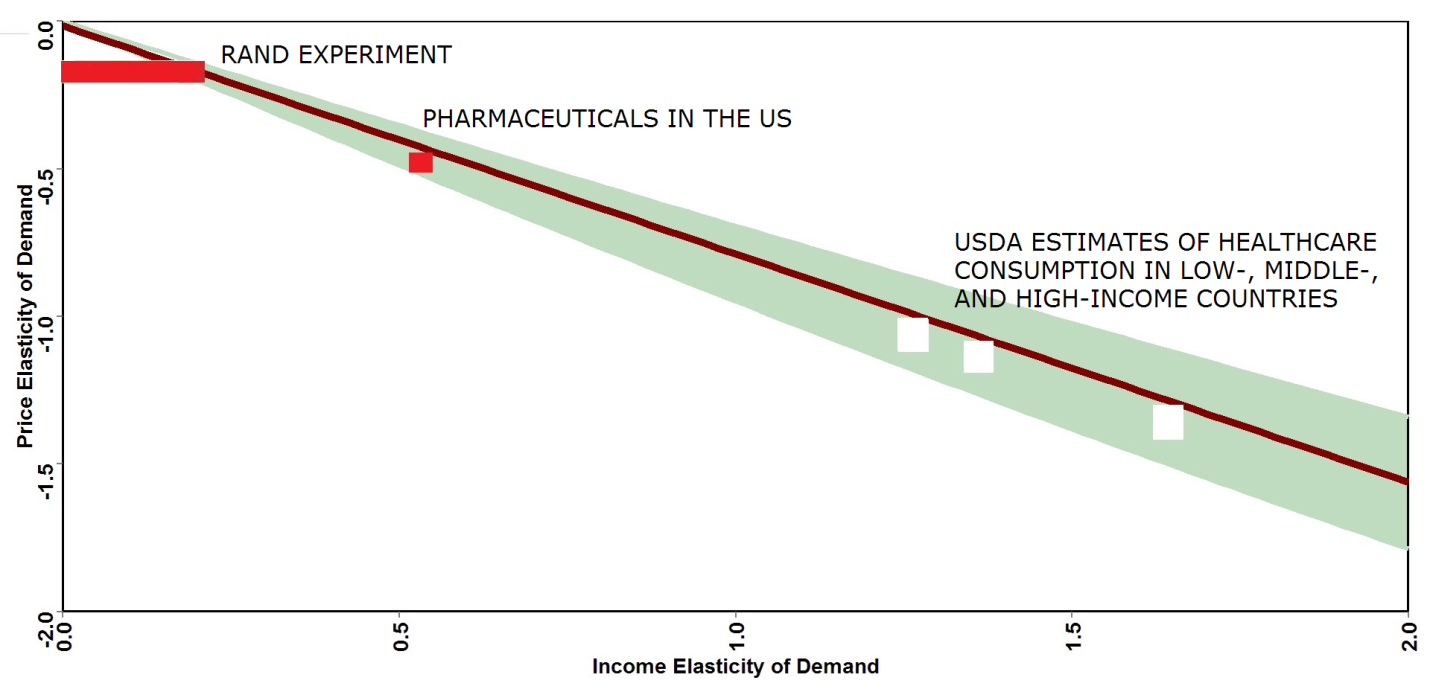


S1.Fig. Comparison between the theoretical relationship and studies that have concurrently assessed income and price elasticity of healthcare. Sources: Santerre & Vernon 2006, Seale et al. 2003, Ringel et al. 2002, & Manning et al. 1987. The green area highlights the 95% credible interval (CrI) of the simulated values of the price elasticity of demand. The red rectangles refer to specific empirical studies conducted in the US. The white rectangles refer to global estimates, based on survey data, produced by the US Department of Agriculture (Seale et al., 2003). The data lying within the 95% CrI is considered consistent with model predictions. The average budget share was drawn from a uniform distribution ranging from 0.0001 to 0.1, and the elasticity of the marginal utility of income was drawn from a normal distribution with mean equal to -1.26 and standard deviation equal to 0.1.
