# Supplementary material for: Relationship between the Uncompensated Price Elasticity and the Income Elasticity of Demand under Conditions of Additive Preferences
Source: PLoS One. 2016 Mar 21;11(3):e0151390. doi: 10.1371/journal.pone.0151390 (PMC4801373; doi:10.1371/journal.pone.0151390)
Supplement: S2 Fig — The colored circles refer to the estimates provided by Seale et al. 2003. The data refer to the following countries: Kenya (low-income); Mexico (middle-income); Italy (high-income). The darker line (in the middle) indicates the median of simulated values, while the lighter external lines define the 95% credible interval calculated using a Monte-Carlo simulation. The average budget share was drawn from a uniform distribution ranging from 0.0001 to 0.1, and the elasticity of the marginal utility of income was drawn from a normal distribution with mean equal to -1.26 and standard deviation equal to 0.1 (DOCX) [file pone.0151390.s002.docx]

**Relationship between Price Elasticity and Income Elasticity of Demand when the Contributions of Goods to the Utility Function are Additive**

Author: Lorenzo Sabatelli, Ph.D

Author affiliation: GLOBMOD Health, Market Analysis Unit, Barcelona, Spain

Correspondence: Lorenzo.Sabatelli@globmod.com

**Supporting figure**


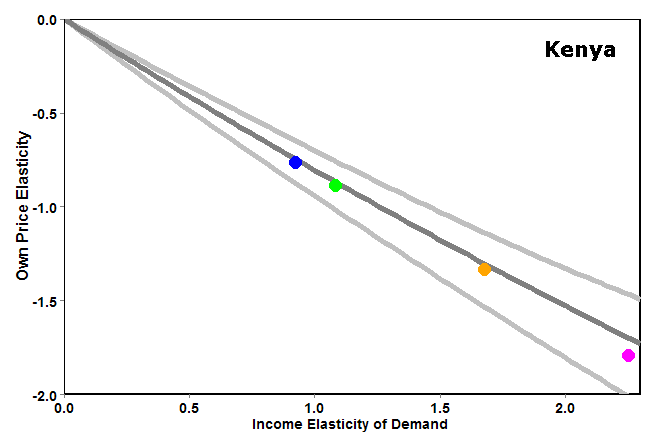

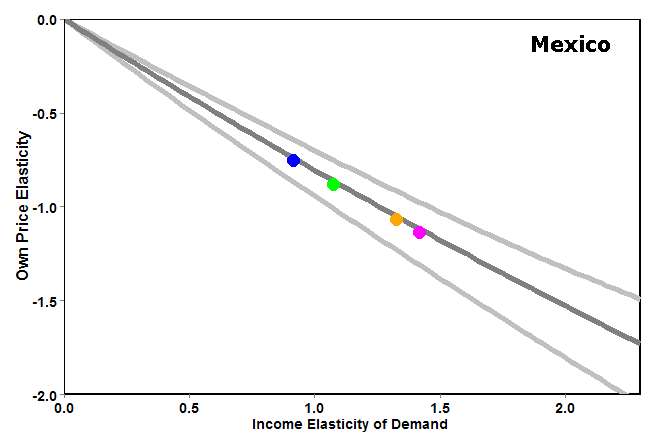

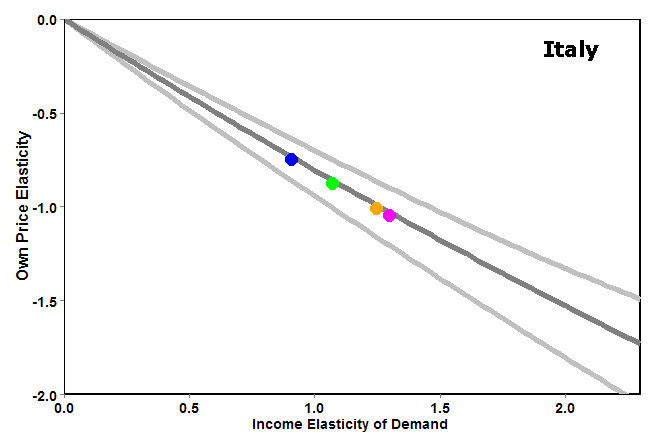

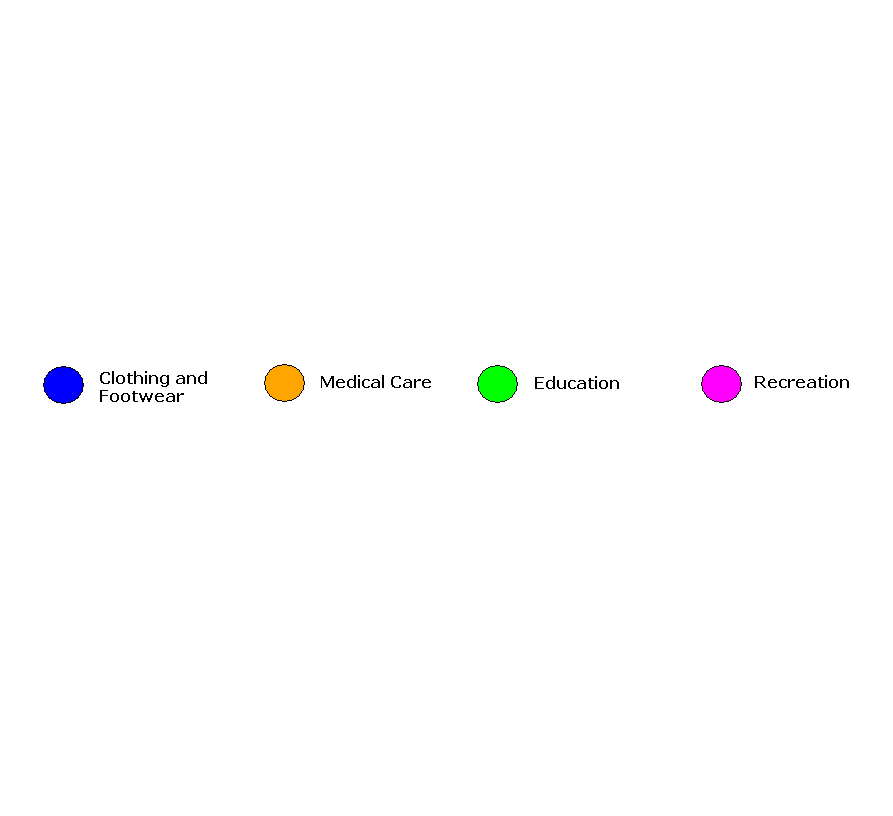


S2.Fig. Comparison of simulation results with estimates of income elasticity and uncompensated own price elasticity of demand for clothing and footwear, education, healthcare, and recreation. The colored circles refer to the estimates provided by Seale et al. 2003. The data refer to the following countries: Kenya (low-income); Mexico (middle-income); Italy (high-income). The darker line (in the middle) indicates the median of simulated values, while the lighter external lines define the 95% credible interval calculated using a Monte-Carlo simulation. The average budget share was drawn from a uniform distribution ranging from 0.0001 to 0.1, and the elasticity of the marginal utility of income was drawn from a normal distribution with mean equal to -1.26 and standard deviation equal to 0.1

.
