# Supplementary material for: Relationship between the Uncompensated Price Elasticity and the Income Elasticity of Demand under Conditions of Additive Preferences
Source: PLoS One. 2016 Mar 21;11(3):e0151390. doi: 10.1371/journal.pone.0151390 (PMC4801373; doi:10.1371/journal.pone.0151390)
Supplement: S1 Text — (DOCX) [file pone.0151390.s003.docx]

**Relationship Between the Uncompensated Price Elasticity and the Income Elasticity of Demand Under Conditions of Additive Preferences**

Author: Lorenzo Sabatelli, PhD

Author affiliation: GLOBMOD Health, Market Analysis Unit, Barcelona, Spain

Correspondence: Lorenzo.Sabatelli@globmod.com

**Supplementary Materials and Methods**

### A simplified proof

The details of the derivation of Eq. (1), and by similarity of Eq. (2), can be better illustrated using a simpler and more intuitive case. Given a market in which only two goods (#1 and #2) are traded, and in which the utility is expressed by the following function:

$$\mathrm{U}\left(q_{1},q_{2}\right)=aq_{1}^{\nu}+bq_{2}^{\tau}\begin{matrix} & \left(A.1\right) \end{matrix}$$

$$\begin{matrix} \begin{matrix} a>0; & b>0; \end{matrix} & \begin{matrix} 0<\nu<1; & 0<\tau<1; & \end{matrix} \end{matrix}$$

with traded quantities $q_{1}$ and $q_{2}$, trading prices $p_{1}$and$p_{2}$, parameters *a* and *b*, and with a fixed total budget *E*, (i.e. $q_{1}p_{1}+q_{2}p_{2}=E$). The following equations must hold if consumers are to maximize their individual utility:

$$\left\{\begin{matrix} \frac{\partial\left[\mathrm{U}\left(q_{1},q_{2}\right)-\mu\left(q_{1}p_{1}+q_{2}p_{2}-E\right)\right]}{\partial q_{1}}=0 \\ \frac{\partial\left[\mathrm{U}\left(q_{1},q_{2}\right)-\mu\left(q_{1}p_{1}+q_{2}p_{2}-E\right)\right]}{\partial q_{2}}=0 \end{matrix}\right.\begin{matrix} & \left(A.2\right) \end{matrix}$$

$$\left\{\begin{matrix} \frac{\partial\mathrm{U}\left(q_{1},q_{2}\right)}{\partial q_{1}}=\mu p_{1} \\ \frac{\partial\mathrm{U}\left(q_{1},q_{2}\right)}{\partial q_{2}}=\mu p_{2} \end{matrix}\right.\begin{matrix} & \left(A.3\right) \end{matrix}$$

where $\mu$ is the marginal utility of income. From which:

$$\frac{\partial\mathrm{U}}{\partial E}=\mu=\nu\frac{a}{p_{1}q_{1}^{1-\nu}}=\tau\frac{b}{p_{2}q_{2}^{1-\tau}}\begin{matrix} & & \left(A.4\right) \end{matrix}$$

$$\frac{\partial^{2}\mathrm{U}}{\partial E^{2}}=\nu\left(\nu-1\right)\frac{a}{p_{1}q_{1}^{2-\nu}}\frac{\partial q_{1}}{\partial E}\begin{matrix} & & \left(A.5\right) \end{matrix}$$

$$\rho=\frac{\frac{\partial^{2}\mathrm{U}}{\partial E^{2}}}{\frac{\partial\mathrm{U}}{\partial E}}E=\left(\nu-1\right)\varepsilon_{1}\begin{matrix} & & \left(A.6\right) \end{matrix}$$

from which:

$$q_{2}=q_{1}^{\frac{1-\nu}{1-\tau}}\left(\frac{\tau}{\nu}\frac{b}{a}\frac{p_{1}}{p_{2}}\right)^{\frac{1}{1-\tau}}\begin{matrix} & & \left(A.7\right) \end{matrix}$$

that substituted in:

$$q_{1}p_{1}+q_{2}p_{2}=E\begin{matrix} & & \left(A.8\right) \end{matrix}$$

yields the equation:

$$E=q_{1}p_{1}+q_{1}^{\frac{1-\nu}{1-\tau}}\left(\frac{\tau}{\nu}\frac{b}{a}\frac{p_{1}}{p_{2}}\right)^{\frac{1}{1-\tau}}p_{2}\begin{matrix} & & \left(A.9\right) \end{matrix}$$

Differentiating Eq. (A.9) with respect to *E*, defining the budget share of good #1 as $\bar{\omega}_{1}=q_{1}p_{1}/E$,

and recognizing that $q_{1}^{\frac{1-\nu}{1-\tau}}\left(\frac{\tau}{\nu}\frac{b}{a}\frac{p_{1}}{p_{2}}\right)^{\frac{1}{1-\tau}}p_{2}=E-q_{1}p_{1}$, yields:

$$\frac{\partial q_{1}}{\partial E}=\frac{q_{1}/E}{\left(\frac{1-\nu}{1-\tau}\right)-\left(\frac{\tau-\nu}{1-\tau}\right)\bar{\omega}_{1}}\begin{matrix} & & \left(A.10\right) \end{matrix}$$

with and the income elasticity for good #1:

$$\varepsilon=\frac{\partial q_{1}}{\partial E}\frac{E}{q_{1}}=\frac{1}{\left(\frac{1-\nu}{1-\tau}\right)-\left(\frac{\tau-\nu}{1-\tau}\right)\bar{\omega}_{1}}\begin{matrix} & & \left(A.11\right) \end{matrix}$$

Differentiating Eq. (A.9) with respect to$p_{1}$, and defining the budget share of good #1 as $\bar{\omega}_{1}=q_{1}p_{1}/E$, yields:

and the uncompensated own price elasticity of demand for good #1:

$$\eta=\frac{\partial q_{1}}{\partial p_{1}}\frac{p_{1}}{q_{1}}=-\frac{\left[\frac{1}{1-\tau}-\frac{\tau}{1-\tau}\bar{\omega}_{1}\right]}{\left(\frac{1-\nu}{1-\tau}\right)-\left(\frac{\tau-\nu}{1-\tau}\right)\bar{\omega}_{1}}\begin{matrix} & & \left(A.13\right) \end{matrix}$$

which is equivalent to Eq. (1), in the main text, in fact substituting Eq. (A.6) and Eq. (A.11) in Eq. (1) yields:

$$\eta=-\frac{1}{\left(\nu-1\right)\varepsilon}\bar{\omega}_{1}\varepsilon^{2}+\left(\frac{1}{\left(\nu-1\right)\varepsilon}-\bar{\omega}_{1}\right)\varepsilon=-\frac{\left[\frac{1}{1-\tau}-\frac{\tau}{1-\tau}\bar{\omega}_{1}\right]}{\left(\frac{1-\nu}{1-\tau}\right)-\left(\frac{\tau-\nu}{1-\tau}\right)\bar{\omega}_{1}}$$

Which is equal to Eq. (A.13).
